# Clinical and phenotypical characteristics of submucosal invasive carcinoma in non-ampullary duodenal cancer

**DOI:** 10.1371/journal.pone.0256797

**Published:** 2021-08-27

**Authors:** Katsunori Matsueda, Hiromitsu Kanzaki, Ryuta Takenaka, Masahiro Nakagawa, Kazuhiro Matsueda, Masaya Iwamuro, Seiji Kawano, Yoshiro Kawahara, Tomohiro Toji, Takehiro Tanaka, Takahito Yagi, Toshiyoshi Fujiwara, Hiroyuki Okada

**Affiliations:** 1 Department of Gastroenterology and Hepatology, Okayama University Graduate School of Medicine, Dentistry and Pharmaceutical Sciences, Kita-Ku, Okayama, Japan; 2 Department of Internal Medicine, Tsuyama Chuo Hospital, Tsuyama, Okayama, Japan; 3 Department of Endoscopy, Hiroshima City Hiroshima Citizens Hospital, Naka-Ku, Hiroshima, Japan; 4 Department of Gastroenterology and Hepatology, Kurashiki Central Hospital, Kurashiki, Okayama, Japan; 5 Department of Practical Gastrointestinal Endoscopy, Okayama University Hospital, Kita-Ku, Okayama, Japan; 6 Department of Diagnostic Pathology, Okayama University Hospital, Kita-Ku, Okayama, Japan; 7 Department of Pathology, Okayama University Graduate School of Medicine, Dentistry and Pharmaceutical Sciences, Kita-Ku, Okayama, Japan; 8 Department of Hepato-Biliary-Pancreatic Surgery, Okayama University Hospital, Kita-Ku, Okayama, Japan; 9 Department of Gastroenterological Surgery, Okayama University Graduate School of Medicine, Dentistry and Pharmaceutical Sciences, Kita-Ku, Okayama, Japan; Campus Bio Medico University, ITALY

## Abstract

**Objective:**

The rare incidence of submucosal invasive non-ampullary duodenal carcinoma has led to scant information in literature; therefore, we compared the clinicopathological features between submucosal invasive carcinoma (SM-Ca), mucosal carcinoma (M-Ca), and advanced carcinoma (Ad-Ca).

**Materials:**

We retrospectively analyzed 165 patients with sporadic non-ampullary duodenal carcinomas (SNADCs) from four institutions between January 2003 and December 2018. The SNADCs were divided to three groups according to histological diagnosis: SM-Ca, M-Ca, and Ad-Ca. The clinicopathological characteristics and mucin phenotypes were compared between groups.

**Results:**

Among the 165 SNADCs, 11 (7%) were classified as SM-Ca, 70 (42%) as M-Ca, and 84 (51%) as Ad-Ca. We found that all SM-Ca (*P* = 0.013) and most Ad-Ca (*P* = 0.020) lesions were located on the oral-Vater; however, an almost equal distribution of M-Ca lesions was found between the oral- and anal-Vater. No significant difference was observed between the tumor diameter of M-Ca and SM-Ca; however, 45% (5/11) of SM-Ca were ≤10 mm. A total of 73% (8/11) of SM-Ca were classified as gastric phenotype and no lesions were classified as intestinal phenotype; whereas most M-Ca were classified as intestinal phenotype (67%, 8/12).

**Conclusions:**

SM-Ca lesions were all located on the oral-Vater and were highly associated with the gastric mucin phenotype, which were different from the features of most M-Ca.

## Introduction

Superficial non-ampullary duodenal epithelial neoplasms (SNADEN) were rare [1,2]; however, most patients presented with the advanced form of the condition, duodenal adenocarcinoma [3,4]. Currently, advancements in endoscopy and the increased use of esophagogastroduodenoscopy as a screening tool have led to the early detection of small SNADENs [5]. Thus, the incidence of mucosal carcinomas (M-Ca) is increasing, whereas that of submucosal invasive carcinoma (SM-Ca) remains low [5,6].

Patients who progress from M-Ca to advanced carcinoma (Ad-Ca) should inevitably develop SM-Ca; therefore, the large discrepancy between the incidences of SM-CA and M-CA or Ad-Ca is contradictory. However, the unavailability of data regarding the relationship of these three conditions has led to poor investigations into the reason. Investigating this relationship will lead to a better understanding of the development and progression of non-ampullary duodenal cancers. Therefore, this study aimed to elucidate the clinicopathological features of non-ampullary duodenal SM-Ca, focusing on the relationship between SM-Ca, M-Ca, and Ad-Ca.

## Materials and methods

### Design and study participants

This was a multicenter, retrospective, observational study conducted at the following four hospitals: Okayama University Hospital, Tsuyama Chuo Hospital, Hiroshima City Hiroshima Citizens Hospital, and Kurashiki Central Hospital.

Prior to initiation of the study, the protocol was approved by the ethics committee of all institutions following the ethics committee of Okayama University Graduate School of Medicine, Dentistry and Pharmaceutical Sciences and Okayama University Hospital on January 4, 2019 (No. 1902–004). The ethics committee of each institution approved the use of the opt-out method to obtain informed consent. The participants had the option to obtain the study information on the onsite information board and website of each institution from January 4, 2019, to December 31, 2020; additionally, they were provided the opportunity to refuse participation. All study data were fully anonymized before they were accessed. All authors approved the final manuscript. This study was performed in accordance with the principles of the Declaration of Helsinki.

This study identified consecutive patients who were histologically diagnosed with duodenal cancer between January 2003 and December 2018. Among them, patients with sporadic non-ampullary duodenal carcinoma (SNADC) were included in this study. Patients with the following characteristics were excluded from the study: (1) tumor located in the ampulla of Vater, (2) invasive cancer from other organs, and (3) familial adenomatous polyposis.

Patients’ medical records were reviewed, and the following clinicopathological parameters were collected: sex, age, primary tumor location and diameter, histological type, mucin phenotype, and Union for International Cancer Control (8th ed.) cancer stage based on tumor, node, and metastasis (TNM) classification. In patients with multiple SNADCs, we evaluated the largest and most advanced lesion. A lesion located at the same level as the ampulla of Vater was categorized as anal-Vater.

### Histological examination

Histological features were evaluated according to the revised Vienna classification of gastrointestinal epithelial neoplasia [7]. Categories 4.2 (carcinoma in situ), 4.3 (suspicious for invasive carcinoma), 4.4 (intramucosal carcinoma), and 5 (invasive neoplasia) were classified as cancer.

In the present study, we classified SNADC into three groups: M-Ca, defined as carcinoma limited to the muscularis mucosae; SM-Ca, as carcinoma invasive to the submucosa; and Ad-Ca, as carcinoma invading the muscularis propria (T2) and deeper (T3 and T4). All lesions were subdivided into differentiated or undifferentiated types depending on histopathological grading. All pathological examinations were performed by qualified pathologists.

### Immunohistochemistry

For all SM-Ca lesions, immunohistochemical examinations were performed using an auto-immunostaining system (Dako EnVision System; Dako Denmark A/S, Glostrup, Denmark). Additionally, a similar protocol was conducted for patients with M-Ca at the Okayama University Hospital which serves as the control group. The mucin phenotype was examined using MUC2 (Ccp58, monoclonal mouse; Leica Biosystems, Newcastle, UK, dilution 1:50), MUC5AC (CLH2, monoclonal mouse; Leica Biosystems; dilution 1:50), MUC6 (CLH5, monoclonal mouse; Leica Biosystems; dilution 1:50), CD10 (56C6, monoclonal mouse; Leica Biosystems; dilution 1:50), and CDX2 (DAK-CDX2, monoclonal mouse; Dako Denmark A/S; dilution 1:50).

### Immunohistochemical evaluation

We performed immunohistochemistry (IHC) for MUC5AC (a marker of gastric foveolar mucin), MUC6 (a marker of gastric pyloric gland mucin), MUC2 (a marker of intestinal mucin), CDX-2 (a marker of intestinal origin), and CD 10 (a marker of the brush border of intestinal epithelial cells). For each marker, positive immunoreactivity was defined as distinct staining in more than 10% of the tumor cells [8].

SNADCs were classified into four types based on the immunohistochemical results. Tumors that were positive only for gastric markers (MUC5AC and MUC6) were classified as gastric type. On the other hand, tumors that were positive only for intestinal markers (MUC2, CD10, and CDX2) were classified as intestinal type. Tumors positive for both gastric and intestinal markers were classified as mixed-type; while tumors negative for both markers were classified as null-type.

Two gastrointestinal pathologists were blinded to the patients’ clinical information and assessed the immunohistochemical results. Any inconsistencies in the IHC assessment were resolved through a final diagnosis decided by a joint assessment.

### Statistical analysis

Continuous variables were represented as median (range) and compared using Wilcoxon’s rank-sum test. On the other hand, categorical variables were represented as frequencies (percentages) and were analyzed using Pearson’s chi-square test or Fisher’s exact test. The Kruskal–Wallis test was used to compare M-Ca, SM-Ca, and Ad-Ca. All tests were two-sided, and statistical significance was set at *P*<0.05. Statistical analyses were performed using the JMP 14 software program (SAS Institute Inc., Cary, NC, USA).

## Results

### Patient and lesion characteristics among M-Ca, SM-Ca, and Ad-Ca

During the study period, 193 patients were histologically diagnosed with duodenal cancer. After exclusion of patients with ampullary tumors (n = 16), invasive cancer from other organs (n = 4), and familial adenomatous polyposis (n = 8), a total of 165 patients with SNADCs were included in the analysis.

The characteristics of patients and lesions are summarized in Table 1. The median age of all patients was 68 years (range, 29–91 years); among the 165 patients, 113 (68%) were men and 52 (32%) were women. Regarding the histological diagnosis, 70 (42%), 11 (7%), and 84 (51%) SNADCs were diagnosed as M-Ca, SM-Ca, and Ad-Ca, respectively. All M-Ca and SM-Ca were evaluated based on histological examination of endoscopically or surgically resected specimens. Among 39 patients with stage II-III Ad-Ca, 36 (92%) were diagnosed using surgically resected specimens. Among 41 patients with stage IV Ad-Ca, 10 (24%) were diagnosed from specimens obtained from palliative surgery. The remaining were diagnosed using biopsy specimens.

**Table 1:** Patient and lesion characteristics among M-Ca, SM-Ca, and Ad-Ca.

	All SNADC *n* = 165	M-Ca *n* = 70	SM-Ca *n* = 11	Ad-Ca *n* = 84	*P*-value*
Sex (male/female) (%)	113/52 (68/32)	47/23 (67/33)	7/4 (64/36)	59/25 (70/30)	0.86
Age, median (range, years)	68 (29–91)	67.5 (36–86)	68 (60–84)	68 (29–91)	0.49
Lesion diameter, median (range, mm)	21 (4–100)	12 (4–60)	12 (6–40)	36 (8–100)	<0.001**
Histology (differentiated/undifferentiated) (%)	144/21 (87/13)	70/0 (100/0)	10/1 (91/9)	64/20 (76/24)	<0.001***
Symptomatic at diagnosis (%)	81 (49)	12 (17)	3 (27)	66 (79)	<0.001****
TNM stage (0-I/II/III/IV)	85/16/23/41	70/0/0/0	11/0/0/0	4/16/23/41	

SNADC, sporadic non-ampullary duodenal carcinoma; M-Ca, mucosal carcinoma; Ad-Ca, advanced carcinoma; SM-Ca, submucosal invasive carcinoma; TNM, tumor, node, metastasis.

*P-value was calculated among M-Ca, SM-Ca, and Ad-Ca.

** M-Ca, SM-Ca vs. Ad-Ca; *p* <0.001, M-Ca vs. SM-Ca: NS.

*** M-Ca vs. SM-Ca: p <0.05, M-Ca vs. Ad-Ca; *p* <0.001, SM-Ca vs. Ad-Ca: NS.

**** M-Ca, SM-Ca vs. Ad-Ca; *p* <0.001, M-Ca vs. SM-Ca: NS.

**Fig 1:**
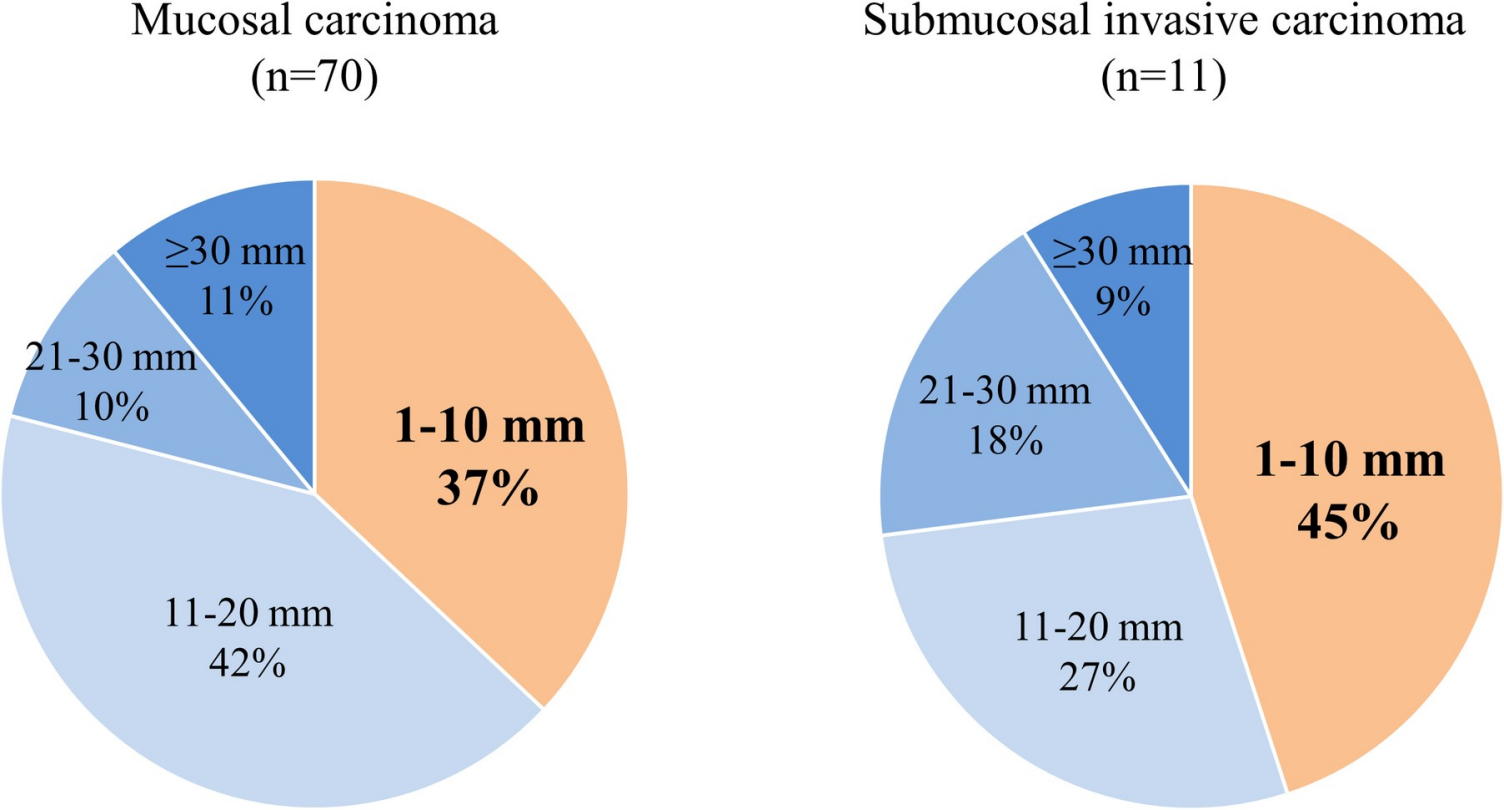
Proportion of mucosal and submucosal invasive duodenal carcinomas according to tumor diameter.

No significant differences were observed regarding the sex or age between the three groups. Large tumor diameter and symptoms were significantly correlated with Ad-Ca (*P* < 0.001); however, this relationship was not observed in M-Ca and SM-Ca. Fig 1 shows the distribution of M-Ca and SM-Ca according to tumor diameter. Although no significant differences were observed in tumor diameter between the two groups, 45% (5/11) of SM-Ca were ≤10 mm in diameter. Regarding histological type, the proportion of undifferentiated-type carcinomas was significantly higher in SM-Ca and Ad-Ca compared to M-Ca (*P* < 0.05, and *P* < 0.001, respectively).

Although no significant differences were observed in tumor diameter between M-Ca and SM-Ca, 45% (5/11) of SM-Ca were ≤10 mm in diameter.

### Comparison of the proportion of SNADC between oral-Vater and anal-Vater

A total of 120 (73%) SNADCs were located on the oral-Vater, and 45 (27%) on the anal-Vater (Table 2). Regarding tumor location of the SNADCs, all SM-Ca lesions were located on the oral-Vater, a higher proportion of Ad-Ca lesions were located on the oral-Vater, and an almost equal distribution between oral- and anal- Vater was observed in M-Ca lesions. The proportion of SNADC on oral Vater was significantly higher in SM-Ca and Ad-Ca compared to M-Ca (100% vs. 61%, *P* = 0.013 and 79% vs. 61%, *P* = 0.020, respectively).

**Table 2:** The proportion of SNADC according to tumor location.

	All SNADC *n* = 165	Oral-Vater *n* = 120	Anal-Vater *n* = 45	*P*-value
M-Ca	70	43 (61)	27 (39)	
SM-Ca	11	11 (100)	0 (0)	0.013*
Ad-Ca	84	66 (79)	18 (21)	0.02**

*P-value was calculated between M-Ca and SM-Ca.

**P-value was calculated between M-Ca and Ad-Ca.

### Comparison of immunohistochemical features between SM-Ca and M-Ca

**Fig 2:**
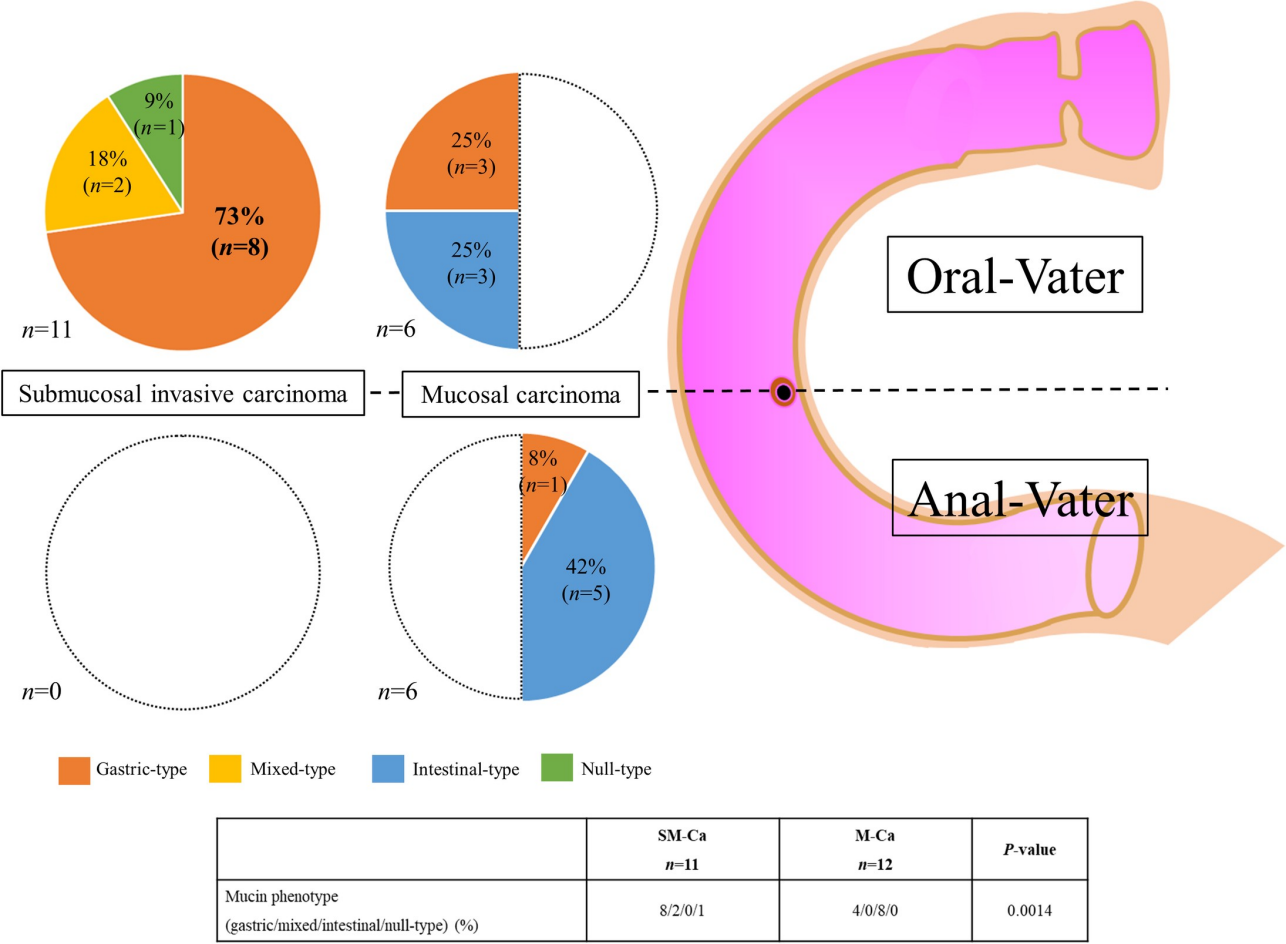
The proportions of mucin phenotype depending on tumor location between submucosal invasive and mucosal carcinoma. Submucosal invasive carcinoma, which was all located on oral-Vater, showed a gastric-type in eight lesions (73%), mixed type in two (18%), and null-type in one (9%). No lesions showed intestinal-type submucosal invasive carcinoma. In contrast, mucosal carcinomas were equally distributed between oral- and anal-Vater; furthermore, intestinal type was observed in eight lesions (67%) and gastric-type in four (33%).

**Fig 3:**
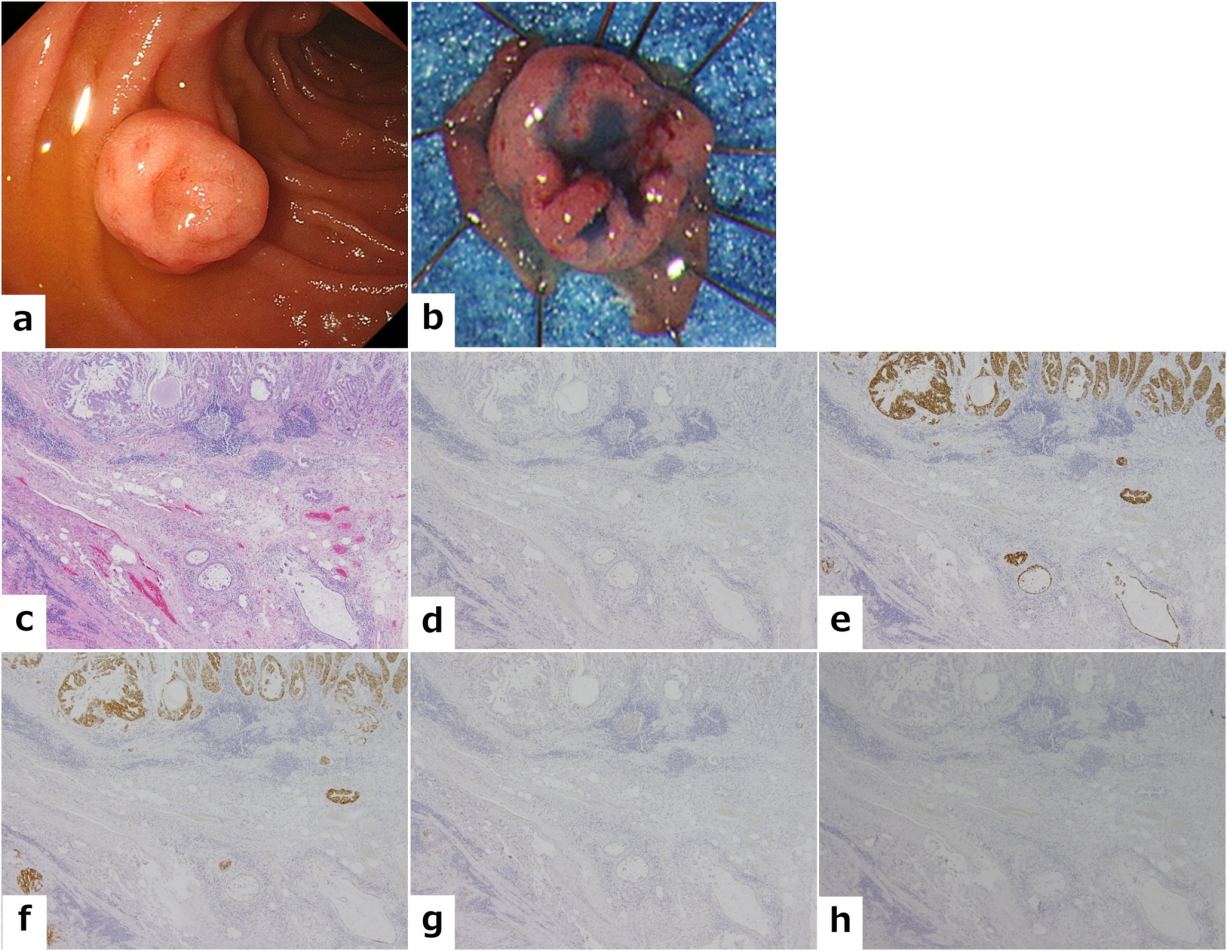
A representative case of a submucosal invasive carcinoma with gastric phenotype on oral-Vater. (a) A 10-mm semi-pedunculated lesion with surface depression is observed on the oral side of the papilla of Vater in the second portion of the duodenum. (b) Macroscopic appearance of the resected specimen shows a clear depression on top of the protrusion. (c) Histological finding shows a well-differentiated adenocarcinoma with submucosal invasion (hematoxylin and eosin stain). Immunohistochemical staining reveals that the tumor cells in both the mucosal and submucosal layer are positive for MUC5AC (e) and MUC6 (f) and negative for MUC2 (d), CD10 (g), and CDX2 (h), revealing a gastric mucin phenotype.

The immunohistochemical features of the mucin phenotype were investigated in all SM-Ca lesions. Twelve consecutive patients with M-Ca from the Okayama University Hospital were designated as the control group. Fig 2 shows the proportions of mucin phenotypes according to tumor location in SM-Ca and M-Ca. A significant difference was observed between the mucin phenotypes of the two groups (*p* = 0.0014). SM-Ca lesions were classified as gastric- (n = 8), mixed (n = 2), and null-type (n = 1), and no lesions were intestinal-type. In contrast, most M-Ca lesions were classified as intestinal-type (n = 8). The proportion of gastric types was higher in SM-Ca compared to M-Ca; however, no significance was found (73% vs. 33%, P = 0.059). The proportion of intestinal-type tumors was significantly lower in SM-Ca compared to M-Ca (0% vs. 67%, P = 0.0008). S1 Table shows the results for clinical features in 11 SM-Ca and 12 M-Ca lesions analyzed by immunohistochemical staining. No significant differences were observed regarding the sex, age, or tumor diameter between the two groups. Fig 3 shows a representative case of SM-Ca with a gastric mucin phenotype on the oral-Vater.

## Discussion

To our knowledge, this is the first study to focus on the clinicopathological features of non-ampullary duodenal SM-Ca in comparison with M-Ca and Ad-Ca. We found a lower incidence of SM-Ca compared to M-Ca and Ad-Ca; additionally, SM-Ca lesions demonstrated the relatively high prevalence of small lesions ≤10 mm in diameter. Furthermore, SM-Ca was strongly correlated with tumor location (anal-Vater) and gastric mucin phenotype, which significantly differed from M-Ca.

In the current study, SM-Ca and M-Ca were significantly different in tumor location; particularly, all SM-Ca lesions were located on the oral-Vater, whereas an almost equal distribution between the oral- and anal-Vater was observed for M-Ca lesions. Previous reports found similarities in the carcinogenic pathway of the adenocarcinoma sequence between duodenal carcinoma and colon cancers [9–11]. Considering this, a similar distribution should be observed between SM-Ca and M-Ca; however, this was not observed in the present study. Therefore, we hypothesized a different etiology or pathway that would explain the difference in distribution between the two lesions.

We found a significantly high association between SM-Ca and gastric phenotype, which was not observed in M-Ca lesions. Exposure to gastric acid can cause the proximal duodenum epithelium to undergo gastric metaplasia in the duodenum (GMD) which results in the gastric phenotype [12,13]. Furthermore, other studies reported that SNADC with gastric phenotype might arise from heterotopic gastric-type epithelium, including GMD and gastric heterotopia (GH), because of their similar genetic alterations [14–16]. These findings implied the role of GMD and GH as potential precursors to SNADC with gastric phenotype [16]. This may explain why most all gastric-type tumors were observed on the oral-Vater in the present study since GMD and GH are often observed in the oral-Vater. The close relationship between gastric-type lesions and the oral-Vater was consistent with previous studies [6,13,17]. Furthermore, most SM-Ca lesions were gastric type, whereas most M-Ca lesions were intestinal type. Gastric phenotype lesions are known to have a high potential to invade deep layers through a diffuse infiltration pattern compared to the intestinal phenotype [18]. This characteristic of the gastric phenotype may be one of the reasons for the stronger likelihood of locating SM-Ca and Ad-Ca on the oral-Vater.

We found that approximately half of SM-Ca lesions had a small diameter of ≤10 mm, which was higher compared to the 37% of M-Ca lesions measuring ≤10 mm; these findings are consistent with previous studies [5,19]. Furthermore, lesion diameter was similar between the SM-Ca and M-Ca groups (12 mm vs. 12 mm in diameter). Submucosal invasion rate of colorectal cancer tends to increase the according to the size via adenoma-carcinoma sequence [20]. Thus, the existence of small SM-Ca was inconsistent with the cancer pathway, as well as the difference in the distribution between SM-Ca and M-Ca.

The differences between SM-Ca and M-Ca regarding tumor location and mucin phenotype, and the presence of small-size SM-Ca imply another cancer pathway of developing lesions located on the oral-Vater, for example, the *de novo* pathway [13]. This pathway is speculated to develop a more aggressive progression compared to the adenoma-carcinoma sequence.

The incidence of Ad-Ca was higher on the oral-Vater compared to the anal-Vater, similar to SM-Ca. Furthermore, the proportion of undifferentiated-type Ad-Ca was significantly higher on the oral-Vater compared to the anal-Vater. Previous studies have reported that differentiated gastric carcinomas with gastric phenotypes demonstrate an increased likelihood to differentiate and behave more aggresively compared to the intestinal phenotype [21]. In this study, most SM-Ca were gastric-type and were located on oral-Vater. In addition, a higher proportion of undifferentiated-type carcinomas was observed in SM-Ca and Ad-Ca compared to M-Ca. These findings suggest that gastric-type carcinomas on the oral-Vater have an increased likelihood of infiltrating the submucosa and differentiating to Ad-Ca. As a result, Ad-Ca have an increased likelihood of developing the oral-Vater, simlar to SM-Ca.

This study has several limitations. First, this was a retrospective study; the design is acceptable considering the rare incidence of SNADC. Second, the mucin phenotype of M-Ca and SM-Ca were discussed; however, the immunohistochemical staining findings of Ad-Ca were unexamined. However, our clinical data clarified that gastric-type superficial SNADC on the oral-Vater have an increased potential of invading the submucosal layer compared to the intestinal-type. Therefore, the present findings might support future research related to the pathogenesis and pathway of SNADC according to the mucin phenotype and tumor location. Third, M-Ca is often difficult to distinguish from high-grade dysplasia based on histological diagnosis [22]. Thus, there might be some cases of high-grade dysplasia in the M-Ca group. Conversely, M-Ca might be under-represented as high-grade dysplasia, which might be excluded from this study. However, we were able to collect a sufficient number of patients with M-Ca for comparison with SM-Ca and showed distinct clinicopathological features between these two groups.

## Conclusions

SM-Ca was predominantly located on the oral-Vater and was highly associated with the gastric mucin phenotype; moreover, approximately half of the lesions were ≤10 mm in diameter, which were different from the features of M-Ca. The discrepancy between the two groups led to the hypothesis of another origin or progression pathway of cancer on the oral Vater. Therefore, additional genomic and molecular studies are required to confirm this hypothesis.

## Supporting information

S1 Table Clinical features of SM-Ca and M-Ca stained by immunohistochemistry. (DOCX)
