# Supplementary material for: Clinical and phenotypical characteristics of submucosal invasive carcinoma in non-ampullary duodenal cancer
Source: PLoS One. 2021 Aug 27;16(8):e0256797. doi: 10.1371/journal.pone.0256797 (PMC8396771; doi:10.1371/journal.pone.0256797)
Supplement: S1 Table — (DOCX) [file pone.0256797.s001.docx]

S1 Table. Clinical features of SM-Ca and M-Ca stained by immunohistochemistry

|  | **SM-Ca**  ***n*=11** | **M-Ca**  ***n*=12** | ***P*-value** |
| --- | --- | --- | --- |
| Sex (male/female) (%) | 8/3　(73/27) | 11/1 (92/8) | 0.23 |
| Age, median (range, years) | 68 (60-84) | 74 (56-86) | 0.81 |
| Lesion diameter, median (range, mm) | 12 (6-40) | 18 (7-30) | 0.39 |
